# Editorial: Retinal Changes in Neurological Diseases

**DOI:** 10.3389/fnins.2021.813044

**Published:** 2022-01-14

**Authors:** Samridhi Sharma, Yuyi You

**Affiliations:** ^1^Faculty of Medicine, Health, and Human Sciences, Macquarie Medical School, Macquarie University, Sydney, NSW, Australia; ^2^Department of Clinical Medicine, Save Sight Institute, University of Sydney, Sydney, NSW, Australia

**Keywords:** retina, optic coherence tomography (OCT), neurodegenerating diseases, imaging, visual pathway, transsynaptic degeneration, non-invasive detection

Evidently, retinal manifestations of structural and functional deterioration are interlinked with the development of neurodegenerative disorders. In addition, the presence of retinal vasculopathy is also tightly linked with cognitive deficits in Alzheimer disease (AD) patients and animal models summarized in a review by Shi et al. These retinal changes are clinically measurable using existing non-invasive techniques such as retinal amyloid imaging, pericyte imaging, optical coherence tomography-angiography (OCT-A), electroretinograms, and fundus imaging, and can be used to monitor disease activity of the brain. This prompts the question of whether monitoring retinal changes (functional, vascular, or structural) *via* non-invasive methods can be routinely deployed for the early diagnosis of neurodegenerative disorders. This theme—early detection of neurodegenerative disease by evaluating structural, functional, and vascular changes in the retina—runs through this issue. Zhang et al. establish that the retinal abnormalities in an AD mouse model (5XFAD) precede the abnormalities in the brain and therefore, could be used for AD diagnosis. The study shows that deposition of amyloid-β plaques leads to thickening of the retina with subsequently reduced light responses of retinal ganglion cells (measured with multielectrode-array recording), which is observed to occur before deterioration in cognitive behavior. Similar retinal pathology reflecting alterations in the brain is seen in Parkinson's disease (PD) in a population-based study conducted by Chen et al. They establish that the patients with PD are at higher risk of retinal diseases at the premotor stage than non-PD controls. However, no significant association was identified between optic nerve disease or glaucoma with PD in this study. The observation of retinal pathology reflecting changes in the brain can help serve as a pre-motor biomarker of PD especially if changes are captured by clinically available non-invasive methods. It was interesting to note in the study by Chen et al. that the effects of PD on retinal pathology were reversed after administration of dopamine supplements warranting a further investigation on the role of dopamine in retina revival and restoration.

In congruence to the correlation between retinal diseases and PD, foveal microvascular alterations are also observed in PD patients. Evaluation of the increasing vascular bed surrounding the foveal avascular zone using OCT-A can help to discriminate PD patients with mild cognitive impairment from controls as established by Murueta-Goyena et al. The result of this study also emphasizes the role of vascular pathophysiology in PD which awaits further exploration. Further, the potential of *in vivo* retinal fundus imaging using OCT to non-invasively evaluate vascular and structural changes along with the alterations of oxygen metabolism can be used to assess multiple sclerosis (MS)-related retinal pathology as shown by Kallab et al. The study establishes oxygen metabolism changes in the retina in MS eyes with a history of optic neuritis (ON), but whether these alterations are disease-specific or occur as a consequence of ON warrants further investigation.

This research topic collection presents the potential of using non-invasive investigation of retinal changes in predicting the onset of neurodegenerative diseases. These retinal changes may occur as a primary pathology or secondary outcome of transsynaptic changes in neurological disorders (Puthenparampil et al., 2017; Asanad et al., 2020; Sharma et al., 2021). The hierarchy of the visual system is linked with one synapse bridging the anterior and posterior ends of the visual pathway, presenting itself as a model that can be clinically monitored for neuro-structural, functional, and vascular changes in the retina reflecting analogous changes in the brain. A comprehensive review published on this subject by our group details existing clinical scenarios showing the transsynaptic changes in the retina that can be measured clinically to detect and monitor the spread of neurodegeneration (Sharma et al., 2021). The simple hierarchy of the visual pathway can help localize lesions in the posterior pathway for clinical differential diagnosis of neurological diseases. The underlying causes of transsynaptic degeneration are however unknown and offer an unexplored avenue for future research. Studies focused on understanding the cellular and molecular mechanisms driving transsynaptic degeneration in the visual system can help unravel the causes and potential therapeutic targets of neurodegenerative diseases. These investigations can be carried out on animal models described in this research topic collection employing the visual system as a model to understand the spread of neurodegeneration, synaptic dysfunction, and the transmission of β-amyloid and tau plaques in the retina from the brain.

In conclusion, advancements in clinical and lab-based imaging equipment offer easy evaluation of structural, functional, and vascular changes in the retina. Several recent articles have reviewed the potential role of novel retinal imaging techniques such as OCT-A and retinal vascular amyloid imaging in monitoring blood flow and metabolism changes in the retina under disease conditions (Gupta et al., 2021; Kashani et al., 2021; Shi et al.). These findings, however, must be validated by using histopathology to establish the specificity and sensitivity of these proposed imaging methods enabling accurate detection. Further, the clinical utility of retinal imaging in most neurodegenerative diseases is based on case studies and from cross-sectional data derived from subjects in advanced disease stages. This needs to be supplemented with large-scale cohort studies to establish the timeline of the changes in the brain and corresponding retinal changes during disease progression. While brain imaging will remain as a standard confirmatory test for the diagnosis of neurodegenerative diseases, retinal imaging has rapidly emerged as a promising clinical tool for non-invasive detection of disease-specific retinal pathology in numerous neurodegenerative disorders in the brain.

## Author Contributions

SS wrote the first draft of the manuscript. YY contributed to manuscript revision, read, and approved the submitted version. Both authors contributed to the article and approved the submitted version.

## Funding

This work was funded by National Multiple Sclerosis Society (Grant no. RG-1907-34571).

## Conflict of Interest

The authors declare that the research was conducted in the absence of any commercial or financial relationships that could be construed as a potential conflict of interest.

## Publisher's Note

All claims expressed in this article are solely those of the authors and do not necessarily represent those of their affiliated organizations, or those of the publisher, the editors and the reviewers. Any product that may be evaluated in this article, or claim that may be made by its manufacturer, is not guaranteed or endorsed by the publisher.
